# Challenges and opportunities in strengthening primary mental healthcare for older people in India: a qualitative stakeholder analysis

**DOI:** 10.1186/s12913-024-10622-y

**Published:** 2024-02-15

**Authors:** Tom Kafczyk, Kerstin Hämel

**Affiliations:** Department of Health Services Research and Nursing Science, School of Public Health, Bielefeld University, Universitaetsstrasse 25, 33651 Bielefeld, Germany

**Keywords:** Aged, Health Services for the Aged, Mental Health, Primary Health Care, Qualitative Research, Health Personnel, General Practice

## Abstract

**Background:**

Primary mental healthcare (PMHC) allows for complex mental health issues in old age to be addressed. India has sought to improve PMHC through legislation, strategies and programmes. This study analyses the challenges and opportunities involved in strengthening PMHC for older persons in India from the perspectives of key stakeholders.

**Methods:**

Semistructured interviews were conducted with 14 stakeholders selected from the PMHC system in India and analysed using thematic analysis. First, the analysis was organizationally structured in accordance with the six WHO mental health system domains: (1) policy and legislative framework, (2) mental health services, (3) mental health in primary care, (4) human resources, (5) public information and links to other sectors, and (6) monitoring and research. Second, for each building block, challenges and opportunities were derived using inductive coding.

**Results:**

This study highlights the numerous challenges that may be encountered when attempting to strengthen age-inclusive PMHC. Among these challenges are poor public governance, a lack of awareness and knowledge among policy-makers and other stakeholders, and existing policies that make unrealistic promises to weak primary healthcare (PHC) structures with an excessive focus on medicalizing mental health problems. Thus, the mental health system often fails to reach vulnerable older people through PHC. Established approaches to comprehensive, family- and community-oriented PHC support attempts to strengthen intersectoral approaches to PMHC that emphasize mental health promotion in old age. Targeting the PHC workforce through age-inclusive mental health education is considered particularly necessary. Experts further argue that adequate monitoring structures and public spending for mental health must be improved.

**Conclusions:**

In this study, we aim to elaborate on the mental healthcare developments that may serve to achieve equity in access to mental healthcare in India. Coordinated and collaborative efforts by public and private stakeholders involved in the care of older persons, both with and without lived mental health experiences, as well as their families and communities, are necessary to bring the vision of those policies for PMHC to fruition. The findings presented in this study can also inform future research, policies and practice in other low- and middle-income countries.

**Supplementary Information:**

The online version contains supplementary material available at 10.1186/s12913-024-10622-y.

## Background

Mental healthcare in low- and middle-income countries (LMICs) has received increasing attention in recent years. For example, the Mental Health Action Plan 2013–2020 of the World Health Organization (WHO) presented an important commitment by governments to prioritize mental health in their public policies [1]. A group that is increasingly recognized as vulnerable to mental health issues in LMICs are older persons [2]. For example, older persons appear as a vulnerable group in the Indian National Mental Health Policy 2014 [3]. This recognition relates to the growing awareness of population ageing in LMICs [4, 5] and of the complexity of mental health issues in old age [6].

Data on China, Ghana, India, Mexico, Russia, and South Africa in the Study on Global AGEing and Adult Health (SAGE) as analysed by Arokiasamy et al. [7] show that, in LMICs, the burden of mental health issues is increasing with age, and the prevalence of mental health impairment is higher in India than in other LMICs. As in most LMICs, among the most prevalent mental health issues in old age in India are depression, anxiety and cognitive impairments [6]. Patel et al. [8] found that 17% of persons aged 60 years and older surveyed in the Indian state of Rajasthan had severe depression, 10.3% had anxiety disorders and 51.2% had cognitive impairments.

In relation to the complexity of mental illness in old age, the positive association between the number of chronic physical diseases and mental health issues must be mentioned [7]. Furthermore, a lower socioeconomic status is associated with lower mental health status in old age [9]. In LMICs, older persons and households with older family members are often socioeconomically deprived. In India, approximately 66% of older persons live below the poverty line and approximately 73% are illiterate [10]. In LMICs, social protection systems, such as pensions and health insurance, often fail to provide adequate protection to older persons [11], placing these individuals at additional risk of mental health issues [12].

### Health system approaches for old-age mental healthcare

Without proper social protection systems, older persons in LMICs often rely on their families for basic necessities [13, 14]. Furthermore, the family plays an important caregiving role in LMICs [15], which is particularly true for India, where traditional values such as filial piety and familialism underpin family care potential [15, 16].

The complex and often chronic mental health needs of older people [6] require multidimensional and diverse responses, including financial, psychological, emotional and social [8]. Such diverse health system approaches and support structures for the mental health and well-being of older persons are needed to avoid overstraining families and to complement already established formal and informal approaches to care. This becomes especially important as family support for older persons is declining in India due to the erosion of traditional family norms [17] and to address the increasing number of older persons living alone [13]. Strong and resilient health systems must focus on the needs and preferences of older persons to address the complex health situations of an ageing population [18]. A key issue is the mental health needs of older persons [18], as emphasized in the Madrid International Plan of Action on Ageing endorsed by the United Nations General Assembly in 2002 [19].

Due to the chronic nature of most mental health issues, health systems should focus on health promotion and disease prevention in addition to long-term care needs [18]. Furthermore, health services should be close to the homes of older persons [20] and should be family- and community-oriented [18]. Primary healthcare (PHC) is seen within this context as a viable, sustainable and strong way forward [20, 21]. The Alma-Ata Declaration of 1978, reinforced by the Astana Declaration of 2018, identified PHC as key to the attainment of complete physical, mental and social well-being [22, 23]. As the first level of care encountered by individuals– the family and community– the PHC approach builds on equity on the basis of need and individual and community empowerment and participation in the planning and implementation of healthcare [22–24]. According to Kringos et al. [25], strong PHC systems incorporate and strengthen the key dimensions of access, continuity, coordination, and comprehensiveness of care. Intersectoral collaboration and interprofessional practices to strengthen and improve PHC services in addressing complex health needs are increasingly emphasized [23, 26, 27].

The integration of mental healthcare at the PHC level, i.e., primary mental healthcare (PMHC), is therefore seen as a critical part of the health system to build a socially just and equitable system that can meet the mental health needs of older persons. Moreover, similar to previous studies on this subject [28, 29], we see mental healthcare for older persons as a cross-cutting issue that spans the old-age, mental health and general healthcare sectors, underlining the need for intersectoral collaboration.

The World Health Organization [30] has summarized each of the key components of a strong mental healthcare system that plays a role in improving mental health. These components are useful to explore the current state, opportunities and challenges of (primary) mental healthcare regarding health system improvements [31]. We use them as a framework to inform our analysis of the challenges and opportunities in strengthening PMHC for older persons in India. They are defined in Table 1.

**Table 1:** Understanding of key components of a mental health system in this study

Key component	Elements and themes
Policy and legislative framework	Policies and legislation, their implementation and the financing of mental healthcare for older persons
Mental health in primary care	Relevance of primary healthcare (PHC), physician and nonphysician-based PHC and interaction with complementary/alternative/traditional health services
Mental health services	Organization of care, specialized mental health services and institutions, psychosocial treatment and psychotropic medicine, equity of access
Human resources	Quantity and quality of human resources and training and educational system for health workers
Public education and links with other sectors	Public education and awareness of mental health in old age, links and collaborations with other sectors such as the social protection sector
Monitoring and research	Monitoring mental health services for older persons and old-age mental health research

Components and elements adopted based on the WHO [32]

### Old-age-inclusive primary mental healthcare (PMHC) in India

In recent years, the need to improve PMHC has been increasingly discussed in Indian society, accompanied by efforts to frame and develop appropriate mental healthcare structures in the public health system. India is in a time of transition towards a broader recognition of the importance of mental healthcare to achieve sustainable development goals [33]. Consequently, measures are focused on realizing the right to access old-age mental health services. For example, the landmark Indian Mental Healthcare Act (MHCA) 2017 gave older persons the right to old-age mental healthcare [28, 34].

Across policies defining the PMHC system in India, such as the National Mental Health Programme [35] or the National Action Plan and Monitoring Framework for Prevention and Control of Noncommunicable Diseases [36], four strategies to better address mental healthcare problems in old age have been identified: (1) integrating community health workers (CHWs) into PMHC, (2) empowering the community to participate in healthcare, (3) supporting the family in a family-led approach, and (4) integrating traditional Ayurveda, Yoga, Naturopathy, Unani, Siddha, Sowa-Rigpa and Homeopathy (AYUSH) services into PMHC [29]. A detailed description of the policies that frame the PMHC system for older persons in India and a contextual description of the architecture of primary mental healthcare (PMHC) in India as envisioned by the policies has been outlined in Kafczyk and Hämel [29].

However, empirical research on the strengths and weaknesses of PMHC in India and envisioned approaches for older persons is largely lacking. In this context, it is necessary to point out that the PMHC system in India is considered to be developed and transformed by public institutions and private (for- and not-for-profit) organizations involved in the process of defining and implementing national legislation, strategies and programmes. Together, they shape the PMHC system and the system’s ability to decisively provide needs-based mental healthcare to older persons. On the national level in India, four key ministries are defining and proposing the way forward for the PMHC system: the Ministry of Health and Family Welfare (MoH&FW), Ministry of Law and Justice (MoLJ), Ministry of Social Justice and Empowerment (MSJE) and Ministry of Ayurveda, Yoga and Naturopathy, Unani, Siddha and Homeopathy (AYUSH) [28]. Private and civil society actors participate in the development of general and mental health policies [28]. Federal-level policies defining the PMHC system call for *“[…] the active participation of several stakeholders including civil society, NGOs, academic and research agencies, development partners, the private sector and, most importantly, the community.”* [37, pp. 2–3], highlighting the complex multistakeholder approach in India for the strengthening and implementation of PHC and mental healthcare herein.

An in-depth exploration at the country level encompassing the macro and micro-level is needed that incorporates different perspectives within the PMHC system to broaden our understanding of this important subject. A current lack of such studies prevents a deeper understanding of the challenges and opportunities for the PMHC system to address the needs of older persons [38–40].

### Aim of the study

Acknowledging that different stakeholders from the public and private spheres shape the mental health system [31], this study’s objective is to analyse challenges and opportunities for the anchoring and implementation of PMHC services for older persons in the health system in India. Our analysis is informed by the WHO’s key components that constitute a strong mental health system ranging from policies to practices. This study helps identify strengths and weaknesses to further develop age-inclusive PMHC in India. By identifying leverage points, these findings could guide further research and the design of policies and practices in India and other LMICs.

## Methods

### Study design

A qualitative study design was employed to analyse challenges and opportunities in the anchoring and implementation of PMHC for older persons as perceived by key national stakeholders in the field. We conducted key informant interviews and analysed them using thematic analyses [41]. The consolidated criteria for reporting qualitative research (COREQ) were followed in the study process [42].

### Sampling and field approach

A key informant (expert) is defined as a person responsible for the development, implementation or control of solutions, strategies, or policies and/or a person with privileged access to policy-level information [43, 44] that pertains to mental healthcare for older persons.

An interdisciplinary perspective approach was adopted [45]. Accordingly, it was our intention to sample different stakeholders involved in framing and developing the (primary) mental health system to represent different perspectives, including federal-level policy-makers or advisors, managers of public and private mental healthcare institutions, representatives from professional psychiatric associations and nongovernmental organizations working on mental health and leading practitioners and researchers on old-age mental healthcare. We believe that our sample goal was reached (see Table 2).

To gain entrance into the field and to obtain a sense of whom to interview, a convenience sample was employed. This was followed by purposive sampling, which was informed by snowballing, a review of the literature, conference papers and a web search. The use of purposive sampling ensured that different perspectives, different practice settings, levels of experience and epistemologies from different stakeholders on the study subject were included. Sampling was conducted by the first author in consultation with the second author.

Our sampling was guided by the following criteria: (1) leading experts in the field of mental health, old-age care and/or primary healthcare, (2) over 10 years of working experiences, (3) involved in national-level policy-making processes and/or the implementation of policies, and (4) good command of English. Notably, the experts were not necessarily those in the highest position of their organization but those with the most expertise and knowledge. The final sample size was determined by saturation, which was interpreted as a matter of not numbers but degree [46].

In total, interviews with 14 key experts from India (5 = female, 9 = male) were conducted and included in this study (see Table 2). To be noted is that most experts in this study were active in multiple areas and were often part of the research field [44].

**Table 2:** Overview of study participants

Expert groups	Description	Interview partners
Representatives of government-affiliated advisory agencies	Leading managers (e.g., policy advisors) from agencies consulting with the federal government on mental health and old-age policy and strategy	3
Researchers	Leading researchers with expertise in mental health, old-age and social care fields	2
Representatives of professional associations	Mental health and geriatric medicine experts (e.g., primary care physicians, psychiatrists, psychologists) practising in public and private primary and secondary care with representative roles in national professional associations	5
Civil society representatives	Managers of large civil society organizations involved in advocating at national-level for older people and working at the community level with older persons with mental health issues	3
Patient representatives	Representatives of national associations for patient self-help groups	1
	**Total**	14

### Key informant interviews

A semi-structured interview guide was developed by the research team (see supplementary material). It covered the following topics: political and public debates and directions in mental healthcare for older persons as well as central challenges and opportunities in PMHC for older persons. The interview-guide consisted of open-ended questions to allow for flexibility during the interviews. However, only through follow-up questions we directed interview partners towards key components of the mental health system, consisting of the policy and legislative framework, mental health within primary care, mental health services, human resources, public education and links with other sectors, and monitoring and research.

To inform the data analysis, at the beginning of each interview, the sociodemographic and professional background of the participants was surveyed. For purposes of anonymity and confidentiality, this information is not presented here. After the first two interviews, the interview guide was reviewed. Since the guide proved to be useful, no adaptations were made. The first author conducted the interviews (median length: 50 min, interquartile range: 26 min). The first author holds a master’s degree in global health, and this study is part of his doctorate studies in public health in Germany (PhD equivalent). Prior to this study, he had worked in the healthcare sector in India. He has 10 years of experience in qualitative research in Germany and LMICs, employing different interview forms and analysis methods. He had no prior relationship with any of the study participants and introduced himself as a researcher. Nonparticipants were not present during interviews. The second author, located in Germany, is the supervisor of the PhD project of the first author, holds a doctorate in social sciences and has comprehensive experience conducting qualitative studies in Germany and abroad for nearly 20 years. All interviews were conducted in English and in person. The language used was English because it allowed the first author (interviewer) to follow the conversation and ask questions, thus facilitating the development of a shared understanding. Challenges posed by a translator could thus be avoided [47]. However, we are aware that subtleties and nuances may be lost when the interviewer and the interviewee are not using their mother tongue. Overall, however, none of the interviews proved to be markedly difficult in terms of language.

It is important to mention that both authors are of non-Indian origin. Data were collected during the first author’s stay of several months in India. The study was discussed and contextualised with different actors from research and practice in India. Being of non-Indian origin was challenging as there was a distinct lack of familiarity with certain Indian concepts and parts of the health system. This familiarity is often developed by persons who grow up in the study context [48]. However, we saw this situation as an opportunity that allowed us to be curious with the unfamiliar, ask taboo questions and be seen as nonaligned with certain subgroups or positions [48]. Since we expected that the non-Indian origin of the primary investigator could influence his positionality on the study subject [49], we continuously and cautiously reflected on our own assumptions to ensure that they did not impact the interpretation of the data and, ultimately, the study results.

### Data collection

Prior to participating in the study, participants received an invitation letter, a subject information sheet and the research was disclosed to them. Hereafter, they either signed an informed consent form or provided verbal consent. Participants were required to give informed consent prior to their participation. Participants were informed that participation was voluntary and they could withdraw from the study at any point without negative consequences.

After consent was obtained from the participants, interviews were recorded, transcribed in their entirety and anonymized. In accordance with applicable data protection rules and regulations and with strict adherence to ethical principles, we maintained the confidentiality of all obtained data. The data were stored on a secured hard drive and were accessible only to the research team.

Interviews were mostly conducted in the offices of the participants (*n* = 10) or in quiet public places (*n* = 4), depending on the participant’s preference. For purposes of confidentiality and privacy, only the interviewer and participant were present during interviews. The interviews took place between 2017 and 2018. This research was delayed because of the coronavirus pandemic.

### Data analysis

We analysed the data based on thematic analysis [50, 51]. Transcripts were first deductively coded using MAXQDA (VERBI GmbH, Plus 2020) by the first author along the organizational codes of the six WHO components for strengthening (mental) health systems: (1) policy and legislative framework, (2) mental health services, (3) mental health in primary care, (4) human resources, (5) public information and links with other sectors, and (6) monitoring and research [32]. After the first transcripts were coded, the first author reviewed whether this approach was feasible or whether adaptations needed to be made; after careful consideration, this approach was followed. In the next step, the material within organizational codes was inductively coded using a spreadsheet to identify themes and relationships. This was an iterative process for the research team where content was grouped and regrouped until each theme could be described comprehensively. During this stage, we also regrouped some themes into other organizational codes that better captured the content. The coding was hence a mix of deductive and inductive coding, sometimes referred to as an integrated approach to coding [52] that organized the data according to its content. We assumed saturation when no new inductively developed themes emerged [53].

## Results

From the data, several themes emerged along the organizational codes and assessment areas of (1) policy and legislative framework, (2) mental health in primary care, (3) mental health services, (4) human resources, (5) public information and links with other sectors, and (6) monitoring and research. These findings are presented in the following. A summary of the organizational codes, themes and initial codes can be found in Table 3.

**Table 3:** Organizational codes, themes and initial codes in this study

Organizational code	Theme	Initial codes
(1) Policy and legislative framework	Slow progress in the political agenda setting	- National discourse on mental health in older age - Attitudes and knowledge of policy-makers - Political space and priorities/recognition of mental health in older age - Policy directions (strategies, programmes, legislation)/formulation of legislative framework - Coordinated policy-making - Voice and participatory policy-making
	Strengthening public health governance for old-age mental healthcare	- Stewardship by the government - Accountability, responsibility of the public health system - Monitoring of governance for old-age mental healthcare - Budget/allocation of resources to mental health for older people - Implementation of policies
	Development assistance: an opportunity or burden?	- Cross-country learning - Influence of international organizations on domestic policy directions - Dependence of India of international (development) organizations - Agenda setting of international organizations
(2) Mental health in primary care	Primary healthcare (PHC): too weak to implement the vision of age-inclusive PMHC?	- Relevance of PHC for old-age mental healthcare - Capacities of the PHC level - Coordination between PHC level and secondary and tertiary care - Integration of mental health (in older age) at the PHC level
	Family- and community-oriented care as an opportunity	- Role of informal care in old-age mental healthcare - Role of community health workers (CHWs) and mid-level care providers - Interplay between informal and formal care
	Integration of traditional health services as part of PMHC	- Role and relevance of Ayurveda, Yoga, Naturopathy, Unani, Siddha, Sowa-Rigpa and Homeopathy (AYUSH) in old-age mental healthcare - Quality of AYUSH practices
(3) Mental health services	Needs-based, comprehensive and collaborative care for older persons: a difficult-to-implement vision	- Needs-based and person-centred care - Comprehensive care - Collaborative and interdisciplinary care - Curative versus preventive care, guiding models of health - Organization of PHC
	Private and public mental health services	- Role of the private sector in old-age mental healthcare - Agenda of the private sector - Emphasis of the private sector on PHC - Role of civil society organization in old-age mental healthcare - Quality of care in the public PHC sector - Collaborative approaches to care between the public and private sector
	Integrating specialized mental health services for older persons into primary healthcare	- Institutional care - Specialized services (e.g., psychotherapy, pharmacotherapy)
(4) Human resources	Shortage of skilled and motivated health workers for old-age-inclusive PMHC	- Skilled human resources - Motivation of human resources - Brain drain and retention factors
	Providing age- and mental health-inclusive education to health professionals	- Specific knowledge of mental healthcare for older persons - Age-inclusive mental health education - Faculties of psychiatry and geriatrics
(5) Public information and links with other sectors	Greater public awareness of mental health in old age is required	- Public awareness of mental health in old age - Awareness-raising programmes
	The social protection sector is important to strengthen old-age mental healthcare and empower older persons	- Social security and protection of older persons - Social welfare programmes - Role of pensions in old-age mental healthcare - Public health insurances for older persons
(6) Monitoring and research	Lack of research on old-age mental healthcare that can translate into practice	- Research on old-age mental health(care) - Monitoring and evaluation of mental healthcare for older people - Data-informed policy development, planning and implementation - Attention to existing research

### Policy and legislative framework

#### Slow progress in the political agenda setting

Interviewees described that the mental health of older persons was long out of the focus of federal and state-level policy-makers. Driven by civil society organizations, such as Alzheimer’s and Related Disorders Society of India (ARDSI) or the Indian Psychiatric Society (IPS), experts now see a broader discourse in India and recognition of the need for improving mental healthcare for older persons at the PHC level. The experts note that discussions should be broadened beyond dementia to better address other impairments, such as depression, psychosis or schizophrenia. An increased interest is reflected in policy developments and implementations, such as the formation of the National Institute of Ageing as foreseen in the National Policy on Senior Citizens (2011).

However, in terms of current policies for old-age mental healthcare, experts are conflicted on whether they meet the current needs of older persons. Interviewees perceive policies as not inclusive of old age and mental health, which is attributed partly to a lack of awareness of this issue and a general lack of coordination and collaboration on a federal level. For example, an indifferent or even discriminatory attitude among policy-makers is described by some experts. Furthermore, according to the experts, current policies for older persons are too medical and institutional and not community-oriented, and they fail to see the need for psychosocial interventions. In a similar way, policy

*[…] focus is the provisions of basic healthcare, for life style diseases, for cardiac, for joint pains for things like that which are more visible more settling diseases rather than this [mental health, added by the authors].* (E11, Civil society representative, Pos. 43)^1^

This is contested by interviewees outlining that new policies reflect a further evolving mental health field for people in older age, highlighting that policy-makers increasingly recognize the importance of old-age mental healthcare and address these needs, as, for instance, in the National Mental Health Policy.

*People are becoming aware that this is a segment which is increasing in number in India now because our life expectancy […]. And they have their own physical and mental health problems. So, the situation has altered very much in favour of them.* (E6, Representative of professional association, Pos. 27)

The process is slow, *“but it is in the right direction. So, we do see things improving in the near future”* (E14, Researcher, Pos. 35). The legislative framework and programs, with the MHCA 2017 and the National Health Programme on Health Care for the Elderly (NPHCE), respectively, mentioned as prominent examples, are described as opportunities to strengthen mental healthcare in the Indian health system, including for older persons. One interview partner optimistically stated,

*I think it is a very progressive legislation [the MHCA 2017, added by the authors]. Very progressive in contrast to our conservative society.* (E9, Representative of professional association, Pos. 35)

What appears to be supported by study participants to strengthen age-inclusive PMHC is the participation of older persons, interest groups and civil society in policy processes.

*[…] older persons are organizing themselves into informal associations […]. So, the need right now is to get together for these old people associations. If they get together, they have a say. And then they can influence government policy, grant making and programs; otherwise […] there is a complete lack of any component of mental healthcare.* (E11, Civil society representative, Pos. 88)

#### Strengthening public health governance for old-age mental healthcare

A major challenge, according to the interviewees, is a lack of accountability and responsibility in the public health system, especially among key ministries. “*Older persons, they keep getting tossed around from here to there. Neither this ministry is doing something for them nor that ministry”* (E7, Civil society representative, Pos. 39). For instance, this makes it difficult for civil society organizations to position the topic of old-age-inclusive PMHC on the political agenda. All interviewed experts demanded more political will, recognition and stewardship by the government:

*I think political will starting right from the top, the recognition by the health ministry, the commitment by health ministers, health secretaries, and then that filters all the way down the system. The recognition that these are chronic diseases […] are the leading causes of illness and disability in old age.* (E2, Researcher, Pos. 46–47)

The expected role of the government to steer and govern should be accompanied by appropriate policies and budgets. Making funding available for mental health for older persons is expected to be challenging, *“[…] the [government official] told that it is very difficult to give resources for elderly people”* (E8, Representative of government-affiliated advisory agency, Pos. 23). For some, this has to do with *“[…] many other things which are competing for the same amount of resources”* (E6, Representative of professional association, Pos. 118–121). Furthermore, data are lacking that would allow to monitor resources invested in the mental health of older people.

*[…] here in public health systems what is the allocation that is given for mental health? Very small. In that, what percentage is going to the elderly mental health? It is very difficult to find out […] it’s not getting identified, reported. And the consequence […] it is difficult for us to sort of make a case to the government. If data is available, you know, it’s very easy for us to look at the allocation.* (E7, Civil society representative, Pos. 17)

The challenges stated above also impact the implementation of policies, which is seen by experts as one of the biggest challenges to better PMHC for older persons. They complain about a lack of implementation capacities at all levels of the Indian health system. For some, this relates to policies that are too idealistic and not drafted in a way to be implementable.

*[…] all these policies are fine you know it talks about a vision. […] But it has to come to the ground, it has to be implemented, somebody needs to execute it.* (E6, Representative of professional association, Pos. 119)

#### Development assistance: an opportunity or burden?

Development assistance as an influencing factor of policies and legislative frameworks appeared to be an important discussion point for experts. Development assistance is discussed as a way to learn from other countries and to bring in experiences and technical know-how to support involved ministries and healthcare actors to be more age- and mental health-inclusive in their policies and programmes and to use resources more effectively. Furthermore, international organizations, such as the WHO, are described as important actors that bring certain topics to the public’s attention; as one expert from civil society stated, “*International pressure makes us do a lot of things. […] When WHO tells you to do something you take it a little more seriously and make some effort to show that we do something. […]”* (E7, Civil society representative, Pos. 39). Some experts demand more pressure from international bodies.

The overall role of development assistance is contested, however. Of concern to experts is that development actors could skew policy directions because they have their own agendas and are less educated about the current needs in India. One expert who was involved in policy processes outlines that *“Development assistance plays a very important role in shaping policy. Most development assistance […] has been damaging to mental health. It has been very harmful in fact because mental health has never been prioritized. […] they are risking skewing our health policy (…)”* (E2, Researcher, Pos. 57). This is contrasted by experts who state that India does not rely anymore on development assistance and can choose in which cases they want to work with development actors.

### Mental health in primary care

#### Primary healthcare (PHC): too weak to implement the vision of age-inclusive PMHC?

The importance of the PHC level for mental health-inclusive care for older persons is not contested by experts. They describe, among other reasons, the advantages of relieving the pressure of secondary and tertiary care and better access for older persons. However, despite recent government efforts to strengthen PHC, most experts see it as a challenge for older persons to find needs-based help at the PHC level for mental health issues– they usually end up at the hospital level, where specialists are available. This is seen by the interviewees as being strongly connected to lacking capacities at the PHC level to provide mental healthcare. The PHC system is described as weak and struggling with systemic problems such as

*[…] desperate shortages of skilled manpower, there are motivational issues […] there is a lack coordination […] If primary healthcare worked with the resources that are already available, I think you would see quite a big change in the quality of care that is being provided. And that would include mental healthcare and care for the elderly.* (E2, Researcher, Pos. 33)

To strengthen mental healthcare at the PHC level, the interviewed experts call for “*the integration of mental health training, mental health education, awareness activities at the primary healthcare and community level.”* Nevertheless, this *“[…] is completely missing”* (E13, Civil society representative, Pos. 52). Some experts underline the importance of integrating mental health into existing structures and programmes for old-age care and warn to not develop new structures that are split from other relevant care sectors for older persons, emphasizing an interdisciplinary approach.

*It is not only dealing with just the mental health things, but it is also dealing with geriatric understanding and seeing the combination of the two.* (E14, Researcher, Pos. 27)

The newly proposed model of Health and Wellness Centres (HWCs)^2^ is linked to optimism in this context among experts as mental health and old-age care services are envisioned to be among the basic services offered here. While the integration of old-age and mental healthcare in HWCs would be “*a distant dream come true to have it”* (E3, Pos. 33), experts close to policy processes are sceptical about whether the government’s concept and implementation strategy is realistic and pertinent to old-age-inclusive PMHC.

#### Family- and community-oriented care as an opportunity

According to the experts, the family plays an important role in caring for older family members with mental health issues and is considered the first level of care: *“We still have the family looking after elderly people. That is the biggest positive which has kept us going so far”* (E3, Representative of professional association, Pos. 41).

If the family is not able to care for older persons with mental health issues, experts highlight the need for community-based care, specifically CHWs and mid-level care providers, to fill that gap. This becomes crucial given the background of declining informal and familial support structures for older persons in India. However, the response to this situation by the formal care sector is perceived to be too slow; moreover, for older persons without much informal support, it is described that.

*[…] there are no formal channels of filling that gap I think that affects their mental well-being a lot. So […] that mental well-being is challenged and there is no response to that, there is no way in which the system tries to identify and respond to it whether formal or informal.* (E7, Civil society representative, Pos. 13)

Most experts believe that CHWs could play a critical role in strengthening community-based PMHC with a focus on mental health promotion and in functioning as a connector between informal and formal care through outreach work. In addition, CHWs could support the family in their caregiving role. However, there are different challenges that need to be overcome, such as the already high workload of CHWs and their traditional focus on communicable diseases and maternal and child healthcare.

#### Integration of traditional health services as a part of PMHC

Traditional health services, specifically AYUSH services, were an often addressed topic by the experts, with most of them pointing out opportunities for PMHC. The interview partners perceived a renewed relevance of AYUSH practices that is related to a push by the government and the media. These practices are highlighted as being more accessible, acceptable and affordable by older persons and are expected to be especially promising for those living in rural areas and/or from lower socioeconomic backgrounds.

Nevertheless, it is also argued that traditional approaches to mental health issues need to be seen with caution, since there *“[…] are lot of unprescribed practices which happen to get rid of psychosis or depression or anxiety which are crack-practices. Those are major challenges in the Indian health scenario”* (E3, Representative of professional association, Pos. 21).

### Mental health services

#### Needs-based, comprehensive and collaborative care for older persons: a difficult-to-implement vision

Most interviewees described person-centred and needs-based, interdisciplinary, comprehensive and collaborative care approaches as important principles to facilitate adequate age-inclusive PMHC. For example,

*[…] a proper mental health setup will require not just a physician, you will require a counsellor, you will require a psychologist, you will require a social worker, you will require in-house therapy unit, medication, you may require ECT [electroconvulsive therapy, added by the authors] or TMS [transcranial magnetic stimulation, added by the authors] therapy.* (E3, Representative of professional association, Pos. 33)

However, there are a number of challenges that result in a *“[…] disparity between the needs of the people and what is being provided by the government”* (E14, Researcher, Pos. 25), which is particularly pronounced in rural areas. According to the experts, the vision of age-inclusive PMHC is far from becoming a reality on the ground: *“Any further improvement will require addressing infrastructural problems, provider issues, those are the system level problems that we need to invest in, not technical fixes”* (E2, Researcher, Pos. 41).

Furthermore, a common challenge is that mental health is often seen under a disease paradigm (*“problem or no problem,”* E1, Pos. 27), which halts the development of comprehensive approaches to care with mental health promotion and prevention as important pillars.

*You know, as a nation, we never realized that mental health is also a part of the overall well-being of the people. So, the focus was more on creating curatives for those illnesses […].* (E13, Patient representative, Pos. 32)

This is seen, for example, in primary care physicians who are not trained in the early detection of mental health issues in older persons. Some experts are pessimistic that this situation will change. To foster comprehensive and collaborative care between different professions, experts demand a stronger integration of the biopsychosocial model of health:

*I see a lot of conflicts in terms of mental health. And reason what I think because of multiple terminologies/aetiologies. Like stakeholders are from different areas. For example, people from psychology, they look at mental health in a different way. People from psychiatry, they are biological people. So, they look at mental health in a different way. People from sociology, they look at mental health in a different way. […] So, the multiple stakeholders are there, and they try to classify, try to address with their own understanding. And that is a major issue. If you try to develop some consensus like bio psycho-social model. If you try to integrate it, well a lot of issues can be resolved.* (E12, Representative of professional association, Pos. 26–27)

Ultimately, *“[…] you have to re-engineer the way primary healthcare itself is organized”* (E2, Researcher, Pos. 49), starting from a person-centred perspective acknowledging that the often chronic nature of mental health problems requires a different health system setup.

#### Private and public mental health services

A common critique of the interviewed experts is that private healthcare providers have gained prominence in mental healthcare: *“Unfortunately, India has seen a lot of privatization in the last 20–25 years, which is not the right trend”* (E5, Representative of professional association, Pos. 81). According to some of the interview partners, the private sector is mostly looking for financial gains, with higher treatment costs and less emphasis on PHC *“[…] because the private sector is not interested in primary healthcare. It’s not a viable area for them”* (E13, Civil society representative, Pos. 54). As a consequence, the private sector fails to provide mental health services for the neediest.

However, *“if we come to the government level (…) it is miserable”* (E1, Patient representative, Pos. 47). Most experts see the need to strengthen the quality of services in the public health sector. Furthermore, it is described that civil society organizations– especially in South India– started to fill care gaps in mental health for older persons and are showing the way with some collaborative approaches between state actors and civil society on a community level. Such collaborative approaches between the state and civil society organizations is seen as a chance to foster age-inclusive PMHC. However, civil society organizations face their own challenges, such as dependency on state actors and difficulties raising funds for older people and mental health, which need to be overcome.

#### Integrating specialized mental health services for older persons into primary healthcare

Some experts see improvements in specialized services for older persons in recent years with an emphasis on clinical levels that could also have the potential to strengthen primary healthcare.

*[…] geriatric clinics have opened in different hospitals which are now providing OPD services and inpatient services where they are dealing with some of these issues [mental health issues, added by the authors].* (E14, Researcher, Pos. 45)

However, the majority underline that *“We don’t have any specific services […] for elderly mentally ill population […]”* (E5, Representative of professional association, Pos. 49), which is particularly the case in rural areas. According to the experts, this needs to be assessed in the context of a lack of any specialized services for older persons and a general inaccessibility of mental health services *“[…] for everyone from childhood to old age”* (E2, Researcher, Pos. 23).

Most experts mention psychotherapy for older persons as an example. It is outlined that psychotherapy is often not a priority of service providers and is barely accessible and affordable in the public sector and accepted by older persons. Experts closer to practical levels highlight as a potential asset of Indian mental healthcare the inexpensive accessibility to psychotropic medication for older persons.

*Like schizophrenia, depression or say any other serious mental illness. One could easily treat for maybe 4 to 5 to 6 dollars in a month. That would be the cost of medication for most of the illnesses. […] which is definitely very less compared to the cost in other countries.* (E5, Representative of professional association, Pos. 71)

### Human resources

#### Shortage of skilled and motivated health workers for old-age-inclusive PMHC

More pronounced in rural areas, a shortage of skilled and motivated health workers is presented prominently by experts as a challenge to more age-inclusive PMHC.

*[…] primary healthcare in India is struggling in many parts of the country. There are desperate shortages of skilled manpower, there are motivational issues, even where there are manpower, they don’t really perform in a kind of way that one would hope.* (E2, Researcher, Pos. 33)

This shortage is seen for a wide range of medical and nonmedical professionals. Some experts specifically highlight the shortage of specialists. One participant who is involved in the organization of community-based services for older persons describes:

*[…] we have not been able to find enough psychologists or neuropsychologists […] if you look at the number of psychiatrists, number of psychological counsellors and they stop at tertiary level. They never go below that level. […] And to take him or her to a rural area would be quite a challenge because if there is one neuropsychiatrist in one particular hospital you don’t have time to go to a rural area.* (E7, Civil society representative, Pos. 21)

This shortage of professionals has been aggravated by brain drain, with many professionals migrating to Europe, the US or Australia, and at the same time, a lack of pulling and retention factors and jobs that attract and keep professionals– especially at the PHC level in rural areas.

*A person who is serving in a small say rural area should be given some privileges. Those privileges are not provided […]. So, resources like their living situation should be better, living facilities may be better, as well as salary structure, facilities for treatment.* (E5, Representative of professional association, Pos. 85)

#### Providing age- and mental health-inclusive education to health professionals

According to the experts, another major challenge is that “*Professionals do not have any concrete knowledge about mental health issues of senior citizens”* (E11, Civil society representative, Pos. 13). For example, *“[…] the primary care physician will not be able to understand that this is a psychiatric disorder”* (E9, Representative of professional association, Pos. 51); this can similarly be seen in that *“[…] the primary care physicians […] have no knowledge of treating psychiatric disorders. […] they all prescribe alprazolam and diazepam, which are restricted. But when it comes to citalopram or other groups, prescriptions are not many”* (E9, Representative of professional association, Pos. 46–47, 49). As a consequence, more cases end up at higher care levels where specialists practice, or the psychosocial problems of older persons and comorbidities are often not treated properly.

Experts demand more age-inclusive education for health professionals that is tailored to the Indian context, so that, for example, psychotherapists *“[…] will learn how to deal with older people”* (E14, Researcher, Pos. 27) and the potential of human resources can be unlocked. If the educational contents of health professionals, including midline health workers and CHWs, are revised to include the mental health of older persons and to be more in line with a biopsychosocial model of health, human resources are regarded as a substantial opportunity to provide better mental healthcare to older persons by experts. In this context, some experts highlight the need to bring closer together the faculties of geriatrics and psychiatry.

*In some part of the country, there is some divisions of geriatric psychiatry. […] those persons who are trained in geriatric psychiatry, of course they can provide more, better services, and they are much more sensitive and aware for geriatric people with mental health illness or issues.* (E12, Representative of professional association, Pos. 33–35)

Improving the knowledge of health professionals of mental healthcare for older people is furthermore seen as a way to reduce what is described as a form of discriminatory attitudes towards mental health and old age. For example, mental health issues, especially among older persons, are often not seen as problems that can be treated by health professionals.

*I don’t think the primary health physician will […] make a diagnosis of a mental health problem. These are considered avoidable. Why waste my time, that’s what they will say, that I have so much work to […], and then what is this mental health?* (E9, Representative of professional association, Pos. 53)

### Public information and links with other sectors

#### Greater public awareness of mental health in old age is required

Experts notably state a lack of awareness and knowledge among the general public– up to the top of the system– of mental health in old age. This is described as a major obstacle towards an age-inclusive PMHC system and reforms thereto.

*There is no public perception […] if you are to go out on the street and ask people what facility would you like the government to open in this country? I am sure nobody will tell you that I want mental health consulting.* (E7, Civil society representative, Pos. 39)

Therefore, experts demand effective awareness programmes with clear messages, such as that treating mental health issues in old age helps.

#### The social protection sector is important to strengthen old-age mental healthcare and empower older persons

Most experts connect the implementation success of PMHC for older persons with the social protection sector. While overall social protection is perceived to improve, there is still a long way to go, according to the interviewees. This is especially important in view of declining informal support structures, a trend that is more pronounced in urban areas.

Pensions are considered important to help create contexts that positively contribute to the mental health of older persons and to make them (financially) more independent of their families, which increases their empowerment. Pensions are described as particularly crucial in the absence of informal support structures.

*If I don’t get pension in old age and I am not employed, then where do I go? Who is worried about my mental health? I am worried about my food and meals. So, both survival questions become very important.* (E7, Civil society representative, Pos. 45)

While experts acknowledge the presence of some pension schemes, access to adequate public pensions for most older persons is challenging. Despite recent movements to improve pension coverage in India, the topic is perceived by experts to be politically difficult.

*And the [government representative, added by the authors] told that it is very difficult to give much resources for elderly people […]; in this government also there is a “no” that they will increase this [for pensions schemes, added by the authors] amount. That is, they are not interested; they are not funding it, like it is a very political and challenging issue.* (E8, Representative of government-affiliated advisory agency, Pos. 21–25)

Public health insurance schemes have been introduced relatively recently as a way to finance the health sector. The legal obligation of insurers as outlined in the MHCA 2017 to provide mental healthcare coverage is perceived by the experts as a long-due development that might, in connection with other social welfare programs, represent a chance to improve the mental health of older people and to improve social justice. Nevertheless, some criticize that PMHC is neglected by current schemes that focus on secondary and tertiary care.

### Monitoring and research

#### Lack of research on mental healthcare for older people that can translate into practice

Regarding research, the experts state a lack of research and data on mental health in old age and appropriate care structures thereto. This lack is more pronounced at lower administrative levels, impedes policy development and the planning and implementation of mental health services at all levels and hinders the monitoring and evaluation of these services and the impact on older persons’ mental health. Therefore, experts closer to the research field suggest investing more time and resources in mental health and mental healthcare research.

At the same time, more public attention needs to be paid to existing research that has been and is being conducted by large, high-level institutions, such as the National Institute of Mental Health and Neurosciences (NIMHANS) or the Institute of Human Behaviour and Allied Sciences (IHBAS).

*They are doing very serious research […]. But, how much of their research is taking public attention and for public policy? You need public attention in the geriatric setup.* (E7, Civil society representative, Pos. 39)

## Discussion

Health systems able to meet the mental health needs of older persons help reduce disability and the financing burden on health systems and contribute to equity in service delivery [18, 19]. In recent national policies in India, such as in the National Mental Health Policy 2014 [3], older persons have been increasingly acknowledged as a group that is vulnerable to mental health problems [28]. In India and other LMICs, PMHC is considered a viable way forward to strengthen mental healthcare for older persons and to address the often complex mental health needs in older age [20, 21]. In our study, we analysed challenges to and opportunities in anchoring and implementing PMHC for older persons in the health system in India from the perspective of key stakeholders; our analysis was organized along the six key WHO components for strengthening mental health systems: (1) policy and legislative framework, (2) mental health in primary care, (3) mental health services, (4) human resources, (5) public information and links with other sectors, and (6) monitoring and research. From an integrated perspective, we present the main conclusions in the following.

### Primary mental healthcare for older persons as a governance issue

Interviewed stakeholders were supportive of what was perceived as a broader discourse in recent years at the federal level on the mental health of older persons and appropriate care structures thereto. However, common areas of criticism demonstrate the need for further changes in the public health system in India to adapt to the demographic and epidemiological transitions [28, 54].

Experts continue to see a lack of awareness and knowledge among policy-makers and other stakeholders as an obstacle to courageous progress in strengthening age-inclusive PMHC (similar for other LMICs: Prince, Livingston and Katona [2]). As it has been suggested earlier, policy-makers and other stakeholders must participate in efforts to raise the understanding of the mental health needs of older persons [17].

Moreover, the experts in this study perceive positive policy developments such as the MHCA 2017 that continued after data collection took place. Since data collection took place, the National Suicide Prevention Strategy has been enacted [55], which can be seen as another positive policy development making suicide prevention a public health priority [56]. Nevertheless, it is unclear how this new strategy contributes to age-inclusive PMHC and warrants further research. Overall, however, the interviewees perceive the actual implementation process as too slow. As shown in this study, major barriers to policy implementation include competing resources, a lack of priority for mental healthcare for older persons, lack of implementation capacities and poor coordination and collaboration among the relevant PMHC actors, or policies that are overly ambitious or not appropriate for implementation. These governance challenges require effective strategies. Petersen et al. [57] identified strategies to strengthen mental health system governance in six LMICs, including in India. These strategies such as the role clarification of different sectors can be applied to the PMHC system in India. In addition, it can be presumed that PMHC is underfinanced [58, 59], which is yet another general barrier to strengthening it. Policy-makers should be aware that a reluctance to increase public spending on mental health casts doubt on establishing a functioning public mental healthcare system (see also: Kafczyk and Hämel [29]). Philip et al. [17] recommended distributing mental health budgets in India specifically for old-age-inclusive mental healthcare and for community-based and facility-based interventions to support the idea of PHC of equity on the basis of need [24].

In consideration of these major structural problems, stakeholders in this study assess that the ambitious policies in India for old-age-inclusive PMHC are not appropriate for implementation. According to the findings of our study, policies should be formulated in a coherent way to enable real changes to follow, e.g., by defining stepwise, short-, middle- and long-term objectives. To foster accountability for the implementation of policies and their progress, adequate monitoring and assessment structures based on transparently available empirical data are needed.

The involvement of mental health service users in policy development and implementation from the top to the bottom of the system has been identified as an important strategy to strengthen inclusive service delivery at the PHC level [60]. This approach is in line with the priorities of policies in India to empower the community to participate in healthcare planning, implementation and monitoring [29]. Similarly, experts in this study suggest involving older persons in policy formulations, their implementations and monitoring. In this context, it was positively highlighted that older persons are organizing themselves more in formal associations. However, the general tenor is that the participation of (representatives of) older persons in healthcare planning and implementation is low. This tenor aligns with other findings from India [28] and other LMICs that have shown that mental health service users are barely involved in strategic decisions [60]. According to those interviewed for this study, a challenge to the increased participation of older persons is the lack of accountability for mental healthcare for older persons among key ministries. Strong public governance structures for age-inclusive PMHC to enable community empowerment through participation are already incorporated into public policies [29], but are insufficiently developed.

In our interview study, representatives from civil society organizations also contemplate their shrinking and restrictive space. Partnerships with and contributions from civil society organizations enable the enrichment of community-based service provision and improve service quality in PMHC; moreover, they are an important source of increased awareness for and support of the self-organization of older persons [61]. Thus, their involvement is advantageous for community empowerment and participation (see: Luisi and Hämel [26]).

The PMHC system is developed and transformed by different public and private stakeholders [28]. Therefore, well-balanced, mixed governance is central to the implementation and performance of health sector programmes [62, 63] and an intersectoral approach to PMHC, which also helps to integrate old-age mental healthcare into existing structures and approaches. This would also be beneficial to overcoming the vertical nature of numerous parts of the health systems and the top-down approach to policy design and implementation, which was alluded to by the experts and previously criticized [29]. This complex multistakeholder approach was also discussed by the interviewees. In this respect, they unanimously criticize India’s mental health strategy as being coined by a liberal agenda that supports a free market. The interviewed experts see a large privatization trend. Private, for-profit organizations in particular tend to provide their services in urban areas and dismiss PHC [64].

In addition, as Kafczyk and Hämel [28] showed, international actors should be mentioned, as they play an important role in policy developments in India. The potential role of development assistance in fostering age-inclusive PMHC is contested by the experts interviewed for this study. They conclude that development actors support learning exchanges between countries and advocate for more old-age-inclusive policy changes. At the same time, development assistance is described as damaging to domestic priorities, as development actors bring their own agendas that barely include mental health. This argument is supported by evidence from Iemmi [65], who found that, globally, international donor funding is not well aligned with mental health needs.

### Primary healthcare orientation and systemic challenges

Stakeholders in this study emphasized the relevance of PHC in the care for older persons’ mental health. According to them, most cases could be treated at the PHC level in India, which is corroborated by the literature outlining that even complex patient cases can be addressed at the PHC level close to the homes of older persons [17, 66, 67]. However, according to experts, the overall response of the health system to mental health still relies strongly on secondary and tertiary care, and policies are perceived as “medical,” “institutional” and are associated with physical health issues. Furthermore, they have a narrow focus on mother and child care, resulting in a disparity between the needs of older persons and the services being provided. These findings are corroborated by a recent policy analysis on age-inclusive PMHC [29]. Experts clearly state that PHC suffers from structural problems, including a lack of equipment. Philip et al. [17, p. 136] argued in this context that PMHC for older persons *“is practically nonexistent in our country.”*

We provide evidence that practitioners refer older persons with mental health problems to higher levels of care, as they lack the capacities to treat such cases. The interplay between PHC and higher levels of care is a known problem in India [29] that needs to be addressed clearly in policies and in practice, e.g., by implementing a functioning gatekeeping role for physicians and integrating specialists such as psychotherapists at the PHC level [13].

Moreover, the findings of our study suggest that mental healthcare for older people in general– and PMHC specifically– is hampered by a shortage of qualified staff. This shortage is more pronounced in rural areas and aggravated by brain drain and a lack of pulling factors. There are no incentives for primary care physicians practising in rural areas. Specialists such as psychologists, neuropsychologists, and geriatric psychiatrists are practising at higher levels and in large institutions, but it stops there. This shortage and maldistribution of staff is well known in India [61, 67, 68] and other LMICs [2]. However, our analyses show that the stakeholders in PMHC perceive human resources as having very large potential in India. A key proposed strategy to unlock this potential is to properly educate health professionals on mental health and mental healthcare for older persons [17, 61, 67]. The curricula for CHWs to specialists need to be reformed to include old-age (mental) healthcare and to bring different fields closer, including geriatric medicine, gerontology and (social) psychiatry and psychology.

Important to consider here is to put some distance from a medical and binary understanding of mental health (i.e., disease and no disease) and move toward a biopsychosocial understanding of (mental) health, which would provide more opportunities to address the social causes of mental health issues in old age and focus on the promotion of mental health. This approach is facilitated by the National Mental Health Policy 2014 [29]. This understanding of (mental) health would also lay the ground for a common framework for different professions, thus facilitating inter- and multidisciplinary approaches to care and overcoming parallel and top-down approaches to service delivery [29]. In a study from rural China, the multidisciplinary, team-based approach comprising village physicians, community workers (referred to as “ageing workers”) and psychiatrists was well approved by healthcare providers to provide integrated care, including mental health promotion [69].

Moreover, age-inclusive training in mental healthcare would provide a way to reduce what stakeholders in this study describe as discriminatory attitudes toward mental health *and* old age. This is supported by evidence from Koschorke et al. [70] for four LMICs, including India, that the training of primary care providers is an opportunity to address stigmatizing beliefs and stereotypes.

Integrated care management approaches in PHC for older persons bringing together mental health, old-age and general health services have shown promising results [71] and have strengthened the continuity of care [17]; for instance, the above cited study in rural China showed that interdisciplinary teams were able to treat not only mental illnesses but also other comorbid conditions, such as hypertension [69]. Experts in this study perceived the new model of HWCs as a potential opportunity to provide integrated care for older persons. A recent study came to a similar conclusion but highlighted that there is still a lack of clarity that needs to be addressed regarding the benefits of HWCs for older persons’ mental health [29].

Integrating traditional care approaches (AYUSH) is perceived as a chance by the interviewed stakeholders, as they are perceived as widely accepted in the older population, particularly in rural regions. These approaches have gained prominence in recent years and are foreseen to be regularly implemented in the proposed HWCs [72]. For Philip et al. [17], this is a chance to develop holistic care for the mental health of older persons. Current policies for mental healthcare for older persons follow this strategy as well and foresee the integration of AYUSH services into PHC centres [29]. Philip et al. [17] further recommended training AYUSH practitioners in geriatric health. Stakeholders in this study nevertheless raise caution regarding the expansion of AYUSH practices. The quality of AYUSH practices and collaboration with the nontraditional health system must be ensured [29, 67, 73].

An important component of PHC is community-based care, and in this context, stakeholders positively outlined that there are more self-organized groups of and for older persons. For instance, there are groups of older persons who engage in laughter therapy together, which has been shown to be an effective activity to reduce depression symptoms [74, 75]. These groups can be seen as a form of peer counselling where people help each other with identifying problems and encouraging each other to adopt healthy lifestyles, leading to improved resilience and social support [76]. Fostering such peer-support groups is a viable way to indirectly promote mental health and enable social networks that can be integrated into a person’s everyday life [13].

Since data collection took place for this study, the coronavirus pandemic has stimulated new, especially digital, ways of providing health and mental health services in India. One example is the National Tele-Mental Health Programme (NTMHP), which provides free round-the-clock mental tele-health services to improve access to mental health counselling and care [77]. It was envisioned that these tele-services would be linked to existing public local mental health services, programmes, and other digital services, such as e-Sanjeevani (a national tele-consultation service) [77]. These services have the potential to improve age-inclusive PMHC [67], but there is still a lack of clarity on how and if this is the case and what challenges and opportunities exist for older persons in India.

Finally, the poor overall social protection for older persons in India [16, 17, 78] makes it crucial to foster an equitable, socially just and (age-)inclusive health system, and access to healthcare without financial hardship is seen by some as a part of PHC [23, 79]. While this was not a focus of this study, we encourage future studies to investigate social protection more closely and how health financing influences older persons’ access to public and private mental healthcare in India. In addition, the importance of stronger social protections is underlined by studies such as Brinda et al. [12], who showed a negative association between social protection coverage and the mental health of older persons in LMICs, including India. Another important reason is that the extended family, which often provides a form of social protection to older family members, is breaking down in India [17].

### Limitations of the study and further research needs

We applied a combination of convenience and purposive sampling, which is prone to selection bias [80]. Other biases may have also interfered with the findings, including interview bias [81] and social desirability bias [82]. English is a common language among experts with national-level influence in India, but conducting interviews only in English meant that non-English-speakers were not sampled. We encourage future studies on the subject to also include non-English-speaking experts.

It should be noted that India is a diverse country with extensive internal heterogeneity, even at the PHC level [83], that is rapidly developing [84]. While different challenges and opportunities exist in different parts of the country, not least because health is a state matter according to the Indian constitution [29], we decided to provide an exploratory, aggregated analysis on the macro-level. Further research that takes a closer look at certain administrative and geographic contexts of the country, including the micro-level which has not been a focus of this study, is needed. We employed a qualitative approach, and future analyses could consider quantitative approaches to validate the findings presented here.

The intention of the study was to explore challenges and opportunities from a systems perspective. We included a multistakeholder sample and were able to provide a broad overview of challenges and opportunities to strengthen the PMHC system in India. Nevertheless, the study was biased towards the provider perspective. More research is needed on the ’fit‘ between the PMHC system and older persons’ needs by using, for example, well-established models of accessibility to care [85]. This would also require including older persons with and without lived experiences with mental health issues, their families, including caregivers, and communities. While we included civil society and patient representative experts in their role as advocates for older persons, this does not replace the inclusion of older persons with and without experiences with mental health issues. Doing so, however, requires a different research approach.

## Conclusions

This qualitative study delineates challenges and opportunities in strengthening primary mental healthcare in India for older persons from the perspective of different stakeholders. The study shows several challenges to old-age-inclusive PMHC that range from weak political governance to insufficient primary care structures. Not only are older people affected by these challenges, but their high level of vulnerability often makes them more dependent on affordable, easily accessible services close to their homes. A main asset for strengthening mental healthcare is that such care could be anchored by drawing on established approaches to comprehensive, family- and community-oriented PHC in India. According to the results of our study, PMHC is a viable way forward for India to achieve equity in access to mental healthcare. However, this is going to be a difficult path to take, as there is no robust network of mental health services at the PHC level in India. It is now time to further build on the mental healthcare developments focused on in this study. Despite the study’s focus on India, the findings presented here can also inform further research, policies and practice in other LMICs confronted with population ageing.

### Electronic supplementary material

Below is the link to the electronic supplementary material.

**Supplementary Material 1:** Semi-structured interview guide

## Data Availability

The qualitative datasets analysed during the current study are not publicly available due to ethical consent restrictions. Upon reasonable request, data extractions can be made available from the corresponding author.

## Abbreviations

AYUSH: Ayurveda, Yoga, Naturopathy, Unani, Siddha, Sowa-Rigpa and Homeopathy
CHWs: Community health workers
HWCs: Health and Wellness Centres
LMICs: Low- and middle-income countries
MHCA: Mental Healthcare Act
PHC: Primary healthcare
PMHC: Primary mental healthcare
WHO: World Health Organization

## Footnotes

1 To improve the readability of direct quotes from the interviews, we have linguistically corrected them where appropriate.

2 Health and Wellness Centres (HWCs) are a key component of ‘Ayushman Bharat– National Health Protection Mission’ and are envisioned as the main mode for the delivery of comprehensive PHC. They are expected to partially replace existing PHC structures, especially Sub Health Centres and Primary Health Centres [37].
