# Supplementary material for: Challenges and opportunities in strengthening primary mental healthcare for older people in India: a qualitative stakeholder analysis
Source: BMC Health Serv Res. 2024 Feb 15;24:206. doi: 10.1186/s12913-024-10622-y (PMC10870524; doi:10.1186/s12913-024-10622-y)
Supplement: Supplementary file 1 — Supplementary Material 1: Semi-structured interview guide [file 12913_2024_10622_MOESM1_ESM.docx]

**Semi-structured interview guide**

*Understanding of mental healthcare*

1. What motivated you to work in mental health? Could you share what mental healthcare is for you and what it entails?

*Characterization of the situation of mental healthcare in India*

1. What is your impression of mental health care globally? How would you characterize the situation of mental healthcare in India?

Let us now take a closer look at the situation of primary mental healthcare for older people in India, for example, the situation of older persons with depression and anxiety

*Central challenges and opportunities in mental healthcare for older persons*

1. What are according to your perspective central challenges in mental healthcare for older people in India?
2. What is going well in mental healthcare in India? What are potential opportunities?

*Political und public directions in mental healthcare for older persons*

1. Is mental health care for older people currently – let’s say in the last five years - debated in India and if yes, could you elaborate on it?
   1. Where is it discussed? What is discussed?
   2. Are more discussions needed? If it should be more discussed, what should be debated in your opinion?
2. Could you tell me (more) about political approaches to mental healthcare for older people in India?

*Challenges and opportunities in service delivery*

1. If we look at the different primary mental healthcare services for older people with mental health issues, which experiences are available and how appropriate are they?
2. If you picture the situation of older persons in India, do you see more or other barriers to care? Do you have examples in mind?
3. Where do you see potential opportunities for improvement in primary mental healthcare?
